# DBS-platform for biomonitoring and toxicokinetics of toxicants: proof of concept using LC-MS/MS analysis of fipronil and its metabolites in blood

**DOI:** 10.1038/srep22447

**Published:** 2016-03-10

**Authors:** Kanumuri Siva Rama Raju, Isha Taneja, Mamunur Rashid, Ashish Kumar Sonkar, Muhammad Wahajuddin, Sheelendra Pratap Singh

**Affiliations:** 1 Pharmacokinetics and Metabolism Division, CSIR- Central Drug Research Institute, Lucknow-226031, India; 2 Academy of Scientific and Innovative Research (AcSIR), CSIR-IITR Campus, Lucknow, India; 3 Analytical Chemistry Laboratory, Regulatory Toxicology and Nanotherapeutics & Nanomaterial Toxicology Group, CSIR-Indian Institute of Toxicology Research, Vishvigyan Bhavan, 31, Mahatma Gandhi Marg, Lucknow 226001, Uttar Pradesh, India

## Abstract

A simple, sensitive and high throughput LC-MS/MS method was developed and validated for quantification of fipronil, fipronil sulfone and fipronil desulfinyl in rat and human dried blood spots (DBS). DBS samples were prepared by spiking 10 μl blood on DMPK-C cards followed by drying at room temperature. The whole blood spots were then punched from the card and extracted using acetonitrile. The total chromatographic run time of the method was only 2 min. The lower limit of quantification of the method was 0.1 ng/ml for all the analytes. The method was successfully applied to determine fipronil desulfinyl in DBS samples obtained from its toxicokinetic study in rats following intravenous dose (1 mg/kg). In conclusion, the proposed DBS methodology has significant potential in toxicokinetics and biomonitoring studies of environmental toxicants. This microvolume DBS technique will be an ideal tool for biomonitoring studies, particularly in paediatric population. Small volume requirements, minimally invasive blood sampling method, easier storage and shipping procedure make DBS a suitable technique for such studies. Further, DBS technique contributes towards the principles of 3Rs resulting in significant reduction in the number of rodents used and refinement in sample collection for toxicokinetic studies.

Fipronil is an insecticide that belongs to the phenyl-pyrazole chemical family. It is used to control cockroaches, ants, beetles, fleas, ticks, termites, rootworms and other insects^1^. Fipronil acts by blocking the GABA_A_-gated chloride channels in the central nervous system which leads to the excessive neuronal stimulation and insect death^1^,^2^,^3^,^4^. Exposure to sunlight degrades fipronil into fipronil desulfinyl in water. Fipronil and its degradation product can bioaccumulate in fish. In comparison to fipronil, fipronil desulfinyl is 9-10 times more active at inhibiting mammalian chloride channel than the insect chloride channel, therefore, reducing the selectivity between insects and humans^1^,^5^,^6^. Metabolism studies in rats and humans have shown that fipronil gets converted primarily into fipronil sulfone, a more persistent metabolite^7^,^8^,^9^. Pharmacokinetic studies in rats suggest that fipronil sulfone persists much longer in circulation than fipronil itself. In rats, the half-life of fipronil and fipronil sulfone were found to be 8.5 and 208 h, respectively^7^. Similar to fipronil desulfinyl, fipronil sulfone is also reported to be twenty times more active at mammalian chloride channels than at insect chloride channels^6^. *In vitro* data in human hepatocytes also indicates that fipronil sulfone is more cytotoxic than fipronil itself^10^. Both fipronil and its sulfone metabolite have shown the potential to cause thyroid dysfunction and increased activity of hepatic enzymes in rats^11^,^12^,^13^. Fipronil has also shown neurotoxic potential in rats and mild nervous trouble in case of acute intoxication in humans^14^,^15^,^16^. Fipronil exposure in humans can occur through domestic and professional uses of fipronil, through food and fish consumption and/or accidental intoxication^11^,^15^,^17^. Biomonitoring of fipronil, its sulfone metabolite and degradation product in different populations is, thus, important for finding their biological exposure and translating the findings of animal studies to humans. Biomonitoring studies involve direct measurement of human exposure to environmental contaminants by measuring such substances or their metabolites in blood, urine, or other specimens and thus, require sensitive and reliable bioanalytical methods of quantification. Few methods have already been reported for determination of fipronil and fipronil sulfone in biological matrices^8^,^18^,^19^. However, these methods utilize tedious and costly techniques of sample preparation, i.e., solid phase extraction, along with long chromatographic run time (>10 min)^8^,^18^. Further, most of the existing methods for biomonitoring studies of environmental contaminants in blood/plasma require relatively the large volume of samples for analysis and the blood is withdrawn by venipuncture which is an invasive technique and requires clinical expertise^8^,^18^.

To the best of our knowledge, for the first time we are reporting a dry blood spot (DBS) approach for the simultaneous estimation of fipronil, fipronil sulfone and fipronil desulfinyl in human and rat blood. This method has a short chromatographic run time of 2 min and utilizes a simple protein precipitation technique for extraction of analytes. Among the many advantages associated with DBS sampling, the ease of samples collection with minimal discomfort and low volume of sample requirement (10 to 20 μL) is of particular importance for biomonitoring studies. Additionally, DBS samples are very convenient to transport and can be collected in home-based settings without the need of medical supervision^20^,^21^. Thus, this novel DBS approach will certainly aid the biomonitoring studies of other environmental toxicants as well. As a proof of concept, we have developed and validated the DBS method for fipronil, fipronil sulfone and fipronil desulfinyl in rat blood as well to expedite the toxicokinetic studies in addition to significantly decreasing the number of animals used. Further, to show the application of the developed method, toxicokinetic study of fipronil desulfinyl following intravenous administration was performed in rats, which is hitherto unreported.

## Results and Discussion

### Optimization of LC-MS/MS condition

**Figure 1:**
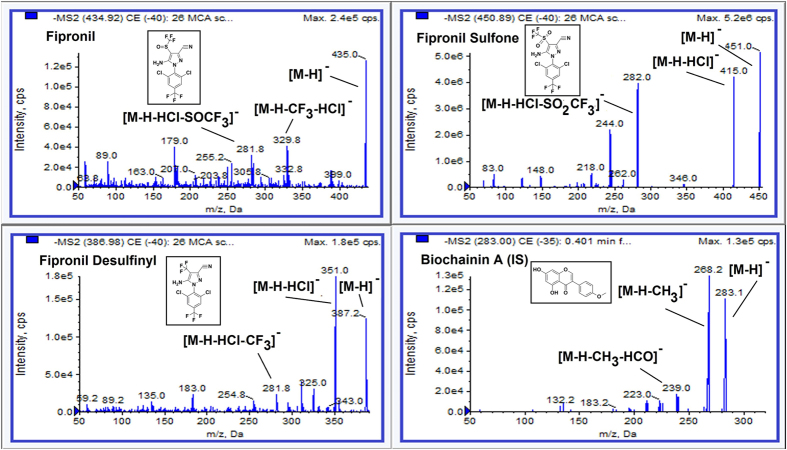
Chemical structures and MS/MS (MS2 scan) spectra of fipronil, fipronil sulfone, fipronil desulfinyl and biochanin A (IS).

Chromatographic conditions and mass related parameters were optimized in order to develop a simple, sensitive and high throughput assay method for quantitation of fipronil, fipronil sulfone and fipronil desulfinyl in rat and human blood. A simple protein precipitation method was adopted using acetonitrile. Acetonitrile provide adequate and reproducible recovery, clean and interference free extract. Different mobile phase, composition and columns were tested to develop a short, robust and sensitive bioanalytical method. Different mobile phases such as ammonium formate, ammonium acetate, formic acid and acetic acid were tested with varying percentage of acetonitrile or methanol as organic modifiers. Waters Atlantis C18 column (4.6 × 50 mm, 5.0 μm) and, acetonitrile and acetic acid (0.1% v/v) in a ratio of 70:30 (v/v) as mobile phase (isocratic mode, flow rate: 0.7 ml/min) offered best sensitivity, peak shape and short chromatographic run time of 2 min. Mass parameters of the fipronil, fipronil sulfone, fipronil desulfinyl and IS were optimized by infusing standard analytes solution (100 ng/ml) prepared in methanol into the mass spectrometer. For optimization of ESI conditions, quadrupole full scans were carried out in both positive and negative ion mode. Negative ion detection mode was found suitable for all the analytes and IS. The full scan (Q1 scan) mass spectra of fipronil, fipronil sulfone, fipronil desulfinyl and IS revealed peaks as protonated ions [M-H]^−^at m/z 434.9, 450.9, 387.0, and 283.0, respectively. Upon product ion scan (MS2 scan), the product ions qualified for monitoring were 329.8 and 281.8 for fipronil, 282.0 and 415.0 for fipronil sulfone, 281.8 and 351.0 for fipronil desulfinyl, and 239.0 and 268.0 for IS. The chemical structures and product ion spectra (MS/MS) of fipronil, fipronil sulfone, fipronil desulfinyl and IS are as shown in Fig. 1. Multiple reaction monitoring (MRM) mass spectrometry conditions based on the better baseline and sensitivity, the precursor ion → product of m/z 434.9 → 329.8 for fipronil, m/z 450.9 → 415.0 for fipronil sulfone, m/z 387.0 → 351.0 for fipronil desulfinyl and m/z 283.0 → 268.0 for IS were used for the quantitation purpose.

### Selectivity

**Figure 2:**
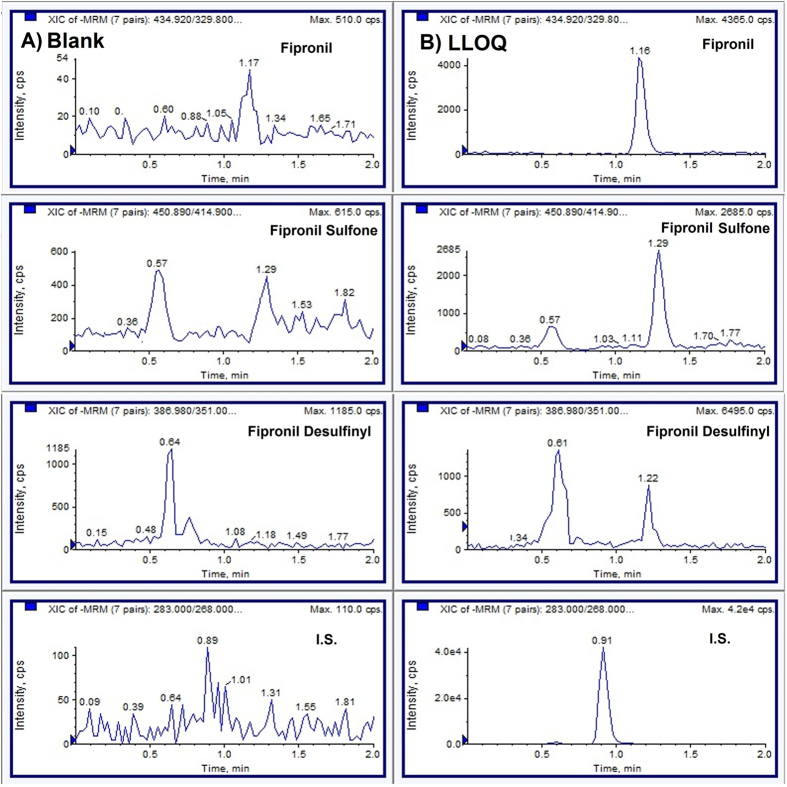
The multiple reaction monitoring chromatograms of (**A**) blank blood (**B**) blood spiked with fipronil, fipronil sulfone and fipronil desulfinyl at LLOQ (0.1 ng/ml), and internal standard (IS, 20 ng/ml). The overall runtime was 2.0 min.

No interfering peaks were observed at the respective retention time (RT) of fipronil (RT: 1.16 min), fipronil sulfone (RT: 1.29) and fipronil desulfinyl (1.22 min) when blank, zero (spiked with IS) and LLOQ sample blood spots were analyzed. Figure 2 shows the chromatograms of blank and LLOQ sample blood spots.

### Linearity, lower limit of quantification (LLOQ) and limit of detection (LOD)

Three measurements of the calibration standards, freshly prepared on three different days, showed good linearity in the range of 0.1 to 100 ng/ml for all analytes. The calibration curves were obtained by selecting the best fit of peak area ratios (peak area analyte/peak area IS) versus concentration and fitted to the y = mx + c using weighing factor (1/X^2^). The correlation coefficient values (r, n = 3) were found to be greater than 0.991 for all the analytes (Table 1). The % accuracy and precision (% RSD) values for the calibration standards including LLOQ were found in the range of 87.67 to 110.33 and 1.33 to 13.44, respectively. These values were in acceptable limits as per the USFDA specifications^22^. The LLOQ of the method for all analytes was 0.1 ng/ml. The LOD of the method was 0.01, 0.01 and 0.03 ng/ml for fipronil, fipronil sulfone and fipronil desulfinyl, respectively (Table 1). A typical chromatogram at the LLOQ is shown in Fig. 2.

Mc Mahen *et al.* analyzed human urine and serum samples for fipronil, sulfone metabolite of fipronil and other possible metabolites using time-of-flight mass spectrometry^8^. No measurable concentrations were found in urine samples for fipronil and its possible metabolites. However, the serum samples showed measurable concentration of fipronil sulfone in the range of 0.1 to 4 ng/ml. Based on these facts; the sensitivity of our method seems suitable for the confirmation of fipronil exposure in humans.

### Extraction recovery and Matrix effect

The recovery and matrix effect were assessed at two concentrations i.e. QC low and QC high (n = 6). The extraction recoveries of fipronil, fipronil sulfone, fipronil desulfinyl and IS were determined in different extraction solvents. Based on the recovery and matrix effect data obtained, the soaking of DBS disc with water (50 μl) followed by extraction with acetonitrile (150 μl) was found to be better extraction solvent. The mean extraction recoveries of all the analytes ranged from 82.49 to 108.23% (Table 2), and the mean extraction recovery of the internal standard was 92.51%.The average matrix effect (ion suppression or enhancement) by blood/paper constituents was less than 10.5% for all the analytes and IS (Table 2).

### Precision and accuracy

The data on intra- and inter-day precision and accuracy of all the analytes are summarized in Table 3. The intra- and inter-day precision showed % RSD values less than 9.95 and 10.05%, respectively for all the analytes in rat and human DBS. The accuracy values of the intra- and inter-day validation were 92.61–108.65 and 95.06–107.31%, respectively. The precision and accuracy values were found to be within the accepted variable limits as per the USFDA^22^.

### Stability studies

The stability assessment of all the analytes on rat DBS samples under different test conditions showed acceptable stability as per the USFDA guidelines for bioanalytical methods^22^. The results are summarized in Table 4. The results revealed that fipronil, fipronil sulfone and fipronil desulfinyl were stable in DBS at room temperature (25 ± 3 °C) for at least 24 h (bench top stability, BT) and 30 days at room temperature (long term stability). All the analytes were found stable in auto sampler for at least 24 h. This information provides the assurance that there will not be any unforeseen stability issues during biomonitoring and toxicokinetic studies of fipronil, fipronil sulfone and fipronil desulfinyl under these tested situations.

### Dilution integrity

**Figure 3:**
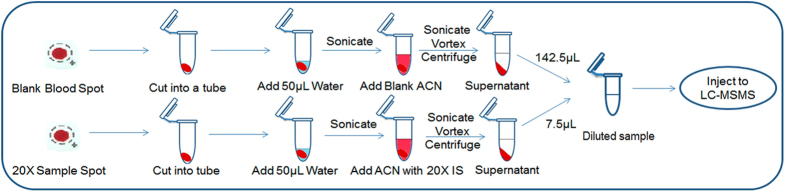
Illustration of dilution integrity process using IS tracked dilution approach for DBS samples analysis.

Dilution of biological matrix is required if study samples concentrations are greater than the upper limit of quantitation of the method (ULOQ, highest point on the calibration curve). Dilution of DBS samples is a major challenge for the bioanalyst due to the solid state of DBS samples. The dilution of DBS samples is not possible in the similar way as for conventional liquid samples such as plasma, urine and serum where samples are diluted with blank matrix and then desired volume used for analysis. In this study, IS-tracked dilution approach was adopted for evaluating the dilution integrity of DBS samples^22^,^23^. The process of the sample preparation for dilution integrity is depicted in Fig. 3. In this approach, the samples to be diluted and regular DBS samples were handled in similar fashion; the only difference was that, the different IS working solution concentration were added to the samples to be diluted. The dilution integrity was investigated at 20 fold dilution factors using the concentration 8 fold greater (800 ng/ml) than the ULOQ. The % precision and accuracy of diluted QCs (n = 4) was in the range of 5.02-10.51 and 89.09-113.52%, respectively for all the analytes. The results suggested that the samples with concentration greater than ULOQ could be re-analyzed by appropriate dilution.

### Toxicokinetic study in rats

**Figure 4:**
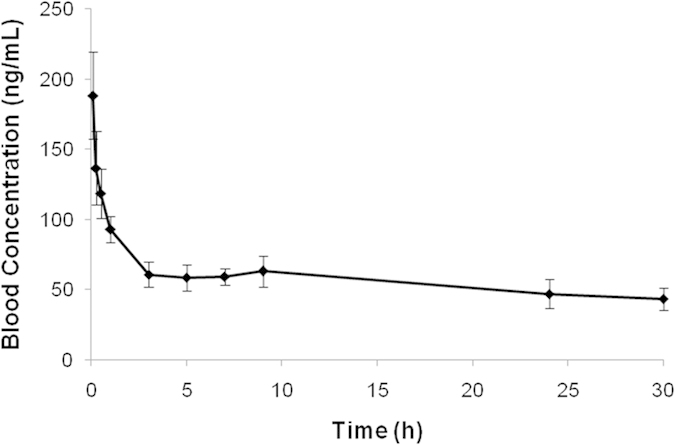
Time course of mean (±SD) blood concentrations of fipronil desulfinyl after intravenous administration in rats at 1 mg/kg.

The toxicokinetics of fipronil and fipronil sulfone already have been reported^13^. However, no toxicokinetic information exists for fipronil desulfinyl in literature. Therefore, we employed this validated method for analyzing toxicokinetic study samples of fipronil desulfinyl conducted in male *Sprague Dawley* rats following intravenous administration at 1 mg/kg dose. The mean blood concentration versus time profile of fipronil desulfinyl is shown in Fig. 4. After intravenous administration, its half-life (t_1/2_) was found to be 36.17 h indicating its slow elimination from the body. A significantly high apparent volume of distribution (V_ss_ = 13.31 ± 2.18 L/kg) than the total body water of rat (0.668 L/kg) suggests that fipronil desulfinyl has a high peripheral distribution into body tissues. The mean hepatic blood flow in rats is 3220 ml/h/kg^24^. The clearance of fipronil desulfinyl was found to be 256.42 ml/h/kg which is ~8% of rat hepatic blood flow indicating that fipronil desulfinyl is low clearance compound. The area under the curve (AUC_0-∞_) of fipronil desulfinyl was found to be 4004.74 ± 732.28 h.ng/ml.

The toxicokinetic study by Roques *et al.* reported the significant fipronil sulfone concentration up to 216 h after the fipronil administration in rats^13^. This suggests that proper toxicokinetic characterization of fipronil metabolite (fipronil sulfone, long half life compound) requires the collection of blood samples for several days. The existing methods for fipronil and fipronil sulfone quantification require minimum ~150 μl of blood volume to get sufficient plasma for sample analysis^18^,^19^. Therefore, it is difficult to collect so many serial samples from same animals without compromising the normal physiological function of the animals. However, our method require only 10 μl blood sample for analysis and hence can easily be adopted to study the exposure and toxicokinetics of fipronil, fipronil sulfone and fipronil desulfinyl in toxicological studies itself without compromising the normal physiology of the animals.

## Materials and Methods

### Chemicals and reagents

Fipronil, fipronil sulfone, fipronil desulfinyl, biochanin A (Internal standard, IS) and formic acid were purchased from Sigma–Aldrich (St. Louis, USA). Acetonitrile and methanol LC-MS grade were purchased from Sisco Research Laboratories Pvt. Limited (Mumbai, India). FTA^®^ DMPK-C blood spot cards, desiccant packets and cutting mats were purchased from GE Healthcare (Gurgaon, India). Milli-Q Ultra-pure water was obtained from a Millipore Elix water purification system purchased from Millipore India Pvt. Ltd. (New Delhi, India). Blank rat blood samples were collected from adult, healthy *Sprague Dawley* rats. Approval from the Animal Ethics Committee of CSIR-Central Drug Research Institute (IAEC/2012/91/NWa/Renew02 (170/14) was taken before the commencement of the studies. Blank human blood samples from healthy human volunteers were collected with their informed consent for making calibration curve and quality control (QC) samples. All the studies were carried out in accordance with the approved guidelines and regulations.

### Preparation of stock solutions, calibration standards and quality control samples

Primary stock solutions of fipronil, fipronil sulfone, fipronil desulfinyl and IS were prepared in methanol at 1 mg/ml. Working standard solutions of fipronil, fipronil sulfone, and fipronil-desulfinyl were prepared by combining the aliquots of each primary stock solution and diluting with methanol to obtain the concentrations of 10 and 1 μg/ml for all the analytes. All the stock solutions were stored at 4 °C until use. The highest concentration (100 ng/ml, highest point on the calibration curve) calibration standard was prepared by spiking stock solution into EDTA containing blank blood of rat and human. A calibration curve was obtained by serial dilution from highest concentration calibration standard to lowest concentration (0.1 ng/ml, lowest point on the calibration curve) calibration standard. Quality control (QC) samples were prepared by individually spiking pooled blank blood at four concentration levels 0.1, 0.3, 40 and 80, ng/ml viz., lower limit of quantitation (LLOQ), QC low (LQC), QC medium (MQC) and QC high (HQC), respectively.

### Blood spotting on DBS card and extraction

10 μl of blank or blood spiked with analytes (calibration standards and quality controls) were spotted onto each printed circle on the DMPK-C cards by the use of calibrated pipette and cards were air dried for 2 h and kept in a sealed plastic bag and stored in the desiccator containing desiccant pack at room temperature 25 ± 3 °C. The relative humidity of the room was 45 ± 5%. For long term stability measurement, DBS cards spiked with QC samples (LQC and HQC) were kept in a sealed plastic bag and stored in the desiccator containing desiccant pack at room temperature 25 ± 3 °C for 30 days. Whole blood spot was punched from each dried blood spot using a punch and transferred to the eppendorf tubes containing 50 μl of MilliQ water. The eppendorf tube was briefly centrifuged to make sure that the DBS disc is completely immersed into the water followed by sonication for 5 min. Then, a 150 μl of acetonitrile with internal standard (20 ng/ml) was added to each sample. In blank sample, no internal standard was added into the acetonitrile. The tubes were vortexed for 2 min and sonicated again for 2 min. The tubes were centrifuged at 10000 rpm for 10 min and 100 μl of supernatant was transferred into the HPLC vial for injection.

### Validation of the assay

The DBS method for quantification of fipronil, fipronil sulfone, fipronil desulfinyl in rat and human whole blood (DBS) was validated according to United States Food and Drug Administration (US-FDA) Bioanalytical Method Validation guidance^22^. The method was validated for selectivity, linearity, limit of detection and lower limit of quantification, intra- and inter-day precision and accuracy, matrix effect, recovery, stability and dilution integrity.

### Selectivity

Potential interference from endogenous compounds was investigated by analyzing the DBS samples prepared from three different human subjects and from six different rat subjects.

### Calibration curve, lower limit of quantification (LLOQ) and limit of detection (LOD)

Calibration curves were made of a blank sample (blood sample spot processed without IS), a zero sample (blood sample spot processed with IS) and non-zero samples (blood sample spot spiked with analytes and IS). The final concentrations of calibration standards were 0.1, 0.2, 0.5, 1, 2, 5, 10, 20, 50 and 100 ng/ml. The LLOQ of the method was defined as the lowest point of the calibration curve. This gave at least ten times the response (a signal to noise ratio > 10) compared to blank. The LOD was calculated by serial dilutions of LLOQ. LOD is the DBS concentration that had a signal to noise ratio ≥ 3.The acceptance criterion for each back calculated calibration standard concentration was 15% deviation from the nominal value except at LLOQ, which was set at 20%.

### Accuracy, precision, recovery and matrix effect

Intra-day accuracy and precision were determined by analyzing six replicates at four different QC levels, viz., 0.1, 0.3, 40 and 80 ng/ml for LLOQ, QC low, QC medium and QC high, respectively. The inter-day assay precision and accuracy were estimated by analyzing the four levels QC samples, six replicates each on three consecutive days (n = 18). Accuracy was evaluated as percentage deviation of the mean from the true value. Precision was expressed as relative standard deviation (RSD) at each QC concentration. The accuracy data to be accepted if the accuracy values were within ± 15% deviation from the nominal concentrations (85–115%), while the precision measured as percentage standard deviation (%RSD) was within ± 15%, except at LLOQ where they can deviate up to ± 20%.

The recovery of fipronil, fipronil sulfone and fipronil desulfinyl were determined at two QC levels, viz., QC low and QC high (0.3 and 80 ng/ml) in six replicates by comparing the areas of extracted DBS samples with those of the post-extraction spiked DBS samples. The matrix effect was determined at two concentrations QC low and QC high (0.3, and 80 ng/ml), by comparing the peak areas of post-extracted DBS samples with peak areas of neat samples^25^. The recovery and matrix effect for biochanin A (IS) was determined at a single concentration 20 ng/ml.

### Stability

The stability of fipronil, fipronil sulfone and fipronil desulfinyl in DBS was evaluated by exposing to different conditions at two QC concentration levels (low and high) in six replicates. The bench top stability of DBS samples was determined at room temperature (25 ± 3 °C) for 24 h. DBS samples for long term stability (30 days) assessment were stored at room temperature conditions in a sealed plastic bag with a small amount of desiccant. Autosampler stability was assessed at 10 °C for 24 h. Samples were considered to be stable if assay values were within the acceptable limits of accuracy (±15%) and precision (±15% RSD), when compared with the freshly prepared QC samples.

### Dilution integrity

Dilution integrity was evaluated by diluting four quality control samples containing 800 ng/ml for all the analytes at 20 fold dilutions using IS tracked dilution method^23^. The process of the sample preparation for dilution integrity is depicted in Fig. 3. For dilution of QCs DBS samples, the DBS samples were mixed with dilution adjusted IS working solution (400 ng/ml IS in acetonitrile for 20 fold dilution) instead of regular IS working solution. For each dilution QC, a double blank DBS (DBLK) sample (blank DBS spot without IS) was prepared in similar fashion. The samples were than extracted as described above. After centrifugation the total volume in both the dilution QCs and DBLK was about 150 μl. Dilution was then done by adding 7.5 μl of the processed dilution QC sample into 142.5 μl of the processed DBLK samples resulting a 20-fold dilution of the processed dilution QCs.

### Toxicokinetic study in rats

Rat *in vivo* toxicokinetic study of fipronil desulfinyl was performed to show the application of the method. Approval for animal experimentation from the Institutional animal ethics committee was sought and all the animal studies were carried out in accordance with the approved guidelines and regulations. Fipronil desulfinyl was administered intravenously at 1 mg/kg dose in male *Sprague Dawley* rats (N = 4, weight range 220 to 240 g). Multiple blood samples (volume ~20 μl) were collected serially from rats into heparinised tubes and 10 μl was spotted on the DMPK-C card. DBS samples were spiked with IS and processed as described above. QC samples along with study samples were processed and distributed among the study samples during analysis. Blood concentration–time data was analyzed by non-compartmental method using WinNonlin Version 5.1 (Pharsight Corporation, Mountain View, USA).

## Conclusion

To our knowledge, this is first DBS method for simultaneous analysis of fipronil, fipronil sulfone and fipronil desulfinyl in rat and human blood using LC-MS/MS. This method utilized simple protein precipitation for sample cleanup. The minimal invasive procedure for sample collection, ease in storage and transport of study samples, very low sample volume requirement for analysis (10 μl) with sensitivity in picogram levels, minimal sample preparation and short analysis run time (only 2 min) makes this method ideal for biomonitoring and toxicokinetic studies. The method was successfully applied to characterize the toxicokinetics of fipronil desulfinyl in rats for the first time. This method can also easily be adopted in forensic laboratories for fipronil–intoxication cases.

## Additional Information

**How to cite this article**: Raju, K. S. R. *et al.* DBS-platform for biomonitoring and toxicokinetics of toxicants: proof of concept using LC-MS/MS analysis of fipronil and its metabolites in blood. *Sci. Rep.* **6**, 22447; doi: 10.1038/srep22447 (2016).

## Figures and Tables

**Table 1:** Parameters for linearity, and values of limit of detection (LOD) and limit of quantitation (LOQ) for fipronil, fipronil sulfone and fipronil desulfinyl in rat and human blood.

Species	Analyte	RT (min)	r ± SD (n = 3)	Slope ± SD (n = 3)	Intercept ± SD (n = 3)	LOD (ng/ml)	LLOQ (ng/ml)
Rat	Fipronil	1.16	0.991 ± 0.006	0.242 ± 0.025	0.163 ± 0.033	0.01	0.1
	Fipronil Sulfone	1.29	0.996 ± 0.003	0.106 ± 0.018	0.036 ± 0.016	0.01	0.1
	Fipronil Desulfinyl	1.22	0.991 ± 0.007	0.015 ± 0.005	0.005 ± 0.003	0.03	0.1
Human	Fipronil	1.16	0.993 ± 0.001	0.267 ± 0.067	0.159 ± 0.018	0.01	0.1
	Fipronil Sulfone	1.28	0.994 ± 0.005	0.134 ± 0.027	0.031 ± 0.024	0.01	0.1
	Fipronil Desulfinyl	1.22	0.996 ± 0.003	0.017 ± 0.002	0.004 ± 0.002	0.03	0.1

**Table 2:** The mean extraction recoveries of fipronil, fipronil sulfone and fipronil desulfinyl from rat and human blood, and matrix effects.

Species	Analyte	Concentration(ng/mL)	Recovery (%)(n = 6)	Matrix Effect(%) (n = 6)
Rat	Fipronil	0.3	94.5	4.5
		80	107.7	8.41
	Fipronil Sulfone	0.3	94.43	2.29
		80	108.23	2.07
	Fipronil Desulfinyl	0.3	85.94	3.32
		80	91.54	6.73
Human	Fipronil	0.3	92.49	10.32
		80	95.72	5.47
	Fipronil Sulfone	0.3	95.43	6.73
		80	102.76	3.48
	Fipronil Desulfinyl	0.3	82.49	7.46
		80	89.51	5.49

**Table 3:** Intra-day (n = 6, at each QC level) and inter-day (n = 18, Six QCs at each level/day for 3 different days) assay precision and accuracy for fipronil, fipronil sulfone and fipronil desulfinyl in rat and human blood.

Species	Compound	Concentration(ng/mL)	aPrecision (RSD)		bAccuracy (%)	
			Intra-day	Inter-day	Intra-day	Inter-day
Rat	Fipronil	0.1	6.02	7.46	94.53	95.06
		0.3	7.93	8.89	94.17	97.93
		40	2.07	10.02	105.79	101.86
		80	2.77	5.95	108.65	107.13
	Fipronil Sulfone	0.1	6.93	7.36	102.88	99.30
		0.3	8.99	7.62	105.39	105.70
		40	3.58	8.25	97.92	96.51
		80	4.34	8.06	93.48	103.73
	Fipronil Desulfinyl	0.1	9.95	9.88	101.03	102.82
		0.3	6.32	9.57	103.78	96.65
		40	3.65	7.33	93.21	99.38
		80	5.67	10.05	98.79	104.34
Human	Fipronil	0.1	6.07	8.78	100.13	98.16
		0.3	5.29	7.37	92.61	95.24
		40	2.60	4.50	97.88	99.82
		80	6.98	5.09	105.27	105.08
	Fipronil Sulfone	0.1	7.31	7.96	100.35	98.22
		0.3	5.28	6.78	103.17	99.61
		40	1.93	3.02	98.38	98.51
		80	2.68	3.23	107.67	107.31
	Fipronil Desulfinyl	0.1	5.34	8.14	101.02	99.57
		0.3	7.45	7.45	105.28	101.67
		40	2.45	4.00	96.42	98.01
		80	4.89	4.84	107.29	107.19

^a^Expressed as % R.S.D. (S.D./mean) × 100.

^b^Calculated as (mean determined concentration/nominal concentration) × 100.

**Table 4:** Stability of fipronil, fipronil sulfone and fipronil desulfinyl in rat blood.

	Fipronil					Fipronil Sulfone				Fipronil Desulfinyl		
	Mean	SD	Precision(%)	Accuracy(%)	Mean	SD	Precision(%)	Accuracy(%)	Mean	SD	Precision(%)	Accuracy(%)
0.3 (ng/mL)												
24 h (AS)	0.29	0.04	12.27	98.16	0.28	0.02	8.28	89.30	0.29	0.03	10.05	105.72
24 h (BT)	0.28	0.02	8.28	93.98	0.28	0.02	8.28	89.30	0.31	0.02	6.32	112.53
30 days at 25 °C	0.31	0.02	7.59	102.34	0.31	0.02	7.66	97.67	0.31	0.03	9.11	112.11
80 (ng/mL)												
24 h (AS)	85.32	7.26	8.51	99.48	85.32	7.26	8.51	99.48	85.58	11.56	13.51	112.88
24 h (BT)	87.73	3.32	3.78	102.29	74.60	3.85	5.17	86.98	79.03	4.48	5.67	104.24
30 days at 25 °C	77.68	5.02	6.47	90.58	89.02	1.86	2.09	103.79	73.85	4.20	5.68	97.41

AS: auto-sampler stability; BT: bench top stability.
